# Liver transplantation in patients with neuroendocrine tumors: a case series and literature review

**DOI:** 10.3325/cmj.2021.62.44

**Published:** 2021-02

**Authors:** Diana Ilić, Nino Kunac, Tina Borčić, Petra Dinjar, Kujundžić, Zrinka Mišetić, Dolić, Nikola Sobočan, Miloš Lalovac, Maja Mijić, Goran Međimurec, Branislav Kocman, Ivana Mikolašević, Tajana Filipec Kanižaj

**Affiliations:** 1 Department of Gastroenterology, Merkur University Hospital, Zagreb, Croatia; 2 Transplant Centre, Department of Surgery, Merkur University Hospital, Zagreb, Croatia; 3 Zagreb University School of Medicine, Zagreb, Croatia

## Abstract

Neuroendocrine tumors (NET) are a rare and heterogeneous group of neoplasms with variable biological behavior. They frequently metastasize to the liver, requiring active, multimodality treatment. Surgical resection, possible in only a minority of cases, was until recently the only potentially curative option. For unresectable NET with liver metastases, liver transplantation (LT) emerged as a potential curative treatment due to relatively slow growth and indolent behavior of the metastases. In this case series with literature review, we retrospectively analyzed the characteristics of 12 highly selected patients with metastatic NET disease as an indication for LT treated in our center. We also summarized the proposed prognostic factors, and evaluated and compared the existing selection criteria. The main poor prognostic factors in our patients were high grade NET and primary tumor in the pancreas. Inconsistent liver transplantation outcome parameters make it difficult to standardize patient selection criteria. There is a need for further studies that would fully elucidate the curative potential of LT in patients diagnosed with NET.

Generally perceived as an indolent, non-aggressive disease, neuroendocrine tumors (NET) comprise a heterogeneous group with variable malignant potential (1-4). These tumors most often arise from the gastrointestinal (60.9%) and bronchopulmonary system (27.4%), with around 50% being functional and producing various hormone-mediated syndromes (1,5). At diagnosis, only 40% of them present as a localized disease. Untreated patients with metastases, mostly in the liver (40%-93%) and bone (12%-20%), experience 20%-40% five-year survival (2,6-9), necessitating a more active therapeutic approach (2,10,11). The pattern of hepatic involvement in most cases is not amenable to curative liver resection (approximately 80% of cases), thus leaving room for various ablative therapies: hepatic artery embolization procedures, peptide receptor radiotherapy, somatostatin analogues, and chemotherapy and molecular-targeted protocols (1,12-15). The relatively slow growing pattern of metastases and their long confinement to the liver make transplantation a reasonable long-term potentially curative option (3,12,14,16,17). Due to disease rarity, heterogeneous tumor parameters, and a lack of clear and prospectively validated patient selection criteria, the transplantation results are highly variable and insufficient to make definitive recommendations (9,17-20).

In this case series with literature review, we retrospectively analyzed the characteristics of 12 highly selected patients with metastatic NET disease as indication for liver transplantation (LT) treated in our center. We critically reviewed pertinent prognostic factors and selection criteria for patients with NET undergoing LT, with an aim to further clarify the true benefit of this complex and potentially curative procedure.

## Case reports

At Merkur University Hospital, the leading liver transplant center in Croatia, 1421 liver transplantations have been performed since 1998. Between 2009 and 2020, 12 patients (0.8% of all liver transplantations) underwent LT due to NET with solitary hepatic metastases (Table 1). They all met the criteria for LT and had previously undergone all oncologic treatment modalities, including primary tumor resection. The criteria for liver transplantation were metachronous metastatic NET disease in the liver without an involved extrahepatic site, time interval between primary tumor resection to LT>12 months, unresectable disease, and portal system drainage of the primary tumor site. All referred patients had undergone LT procedure. Cases were non-consecutive. Six women and six men were included (median age of 44.33 years, range 34-54). Endocrine activity was noted in three patients (0.25%) with elevated chromogranin A and 5-hydroxyindoleacetic acid. The primary tumor location was the pancreas (7 patients – 58.3%), liver (1 patient – 8.3%), liver graft (donor-origin NET, 1 patient – 8.3%), retroperitoneum (1 patient – 8.3%), small intestine (2 patients – 16.6%). The primary tumor was resected in nine patients, along with liver metastases resection and radiofrequency ablation in one patient, and resection was not performed in two patients with primary tumor location in liver/liver graft. In the pretransplantation period, four patients were treated with somatostatin analogue and three received chemotherapy consisting of cisplatin and etoposide. One patient was given somatostatin analogue and chemotherapy. Two patients did not receive any preoperative therapy. The time between the diagnosis of liver metastases and LT ranged between 11 and 145 months (median, 42.3 months), and in two patients with primary NET in liver/liver graft there was no time delay. Eleven patients underwent orthotopic LT, with classic vascular and biliary anastomoses, without any additional resection. A living donor liver transplantation was performed in one patient as a life-saving procedure due to hepatic artery thrombosis. Ki67 proliferation index analysis of the overall explanted tumor tissue did not exceed 24%. Out of 12 patients, two had Ki67 > 20%, both of them having a recurrent disease (100%), while among 10 patients with Ki67 < 20% only two had a recurrent disease (20%).

**Table 1:** Patients with neuroendocrine tumors who underwent liver transplantation in Merkur University Hospital from 2009 to 2020

Patient's number	Age at transplantation	Sex	Primary tumor location	Functional status	Ki-67, %*	Tumor grade^†^	Pretransplantation therapy	Primary tumor to transplantation (months)	Recurrence (months)	Year of transplantation	Post transplantation follow-up (months)
1	54	F	pancreas	no	4	G2	octreotide	14	no	2017	33
2	36	M	pancreas	yes	6	G2	cisplatin + etoposide	43	yes (17)	2014	60
3	38	F	pancreas	no	<5	G2	octreotide	42	no	2018**	13
4	63	F	ileum	no	4	G2	dacarbazine + epirubicin 5-fluorouracil	15	yes (17)	2018	20
5	59	M	pancreas	no	23	NEC-G3	no	15	yes (21)	2017^II^	31
6	45	M	pancreas	no	<10	G2	no	72	no	2015	60
7	44	F	pancreas	no	24	NEC-G3	cisplatin + etoposide,octreotide	1	yes (48)	2015	54
8	34	F	liver	yes	3	G2	octreotide	0	no	2009	130
9	64	M	pancreas	yes	1	G1^‡^	radiofrequency ablation and chemotherapy regimen (cisplatin + etoposide)	11	no	2012	83
10	48	F	retroperitoneum	no	10	G2	octreotide	132/18^§^	no	2014	66
11	47	M	liver graft^††^	no	16	G2	no	/	no	2018^‡‡^	8^¶^
12	41	M	small intestine	no	9	G2	octreotide	20	no	2019	2 (died)

*performed on explanted liver tissue.

†diagnosis defined after insight in explanted liver tissue.

‡insulinoma.

^§^extirpation of primary tumor/extirpation of recurrent tumor.

IIliver + pancreas.

^¶^after second transplantation.

**retransplantation for hepatic artery thrombosis.

^††^primary tumor: donor lungs.

^‡‡^year of the second liver transplantation.

**Figure 1:**
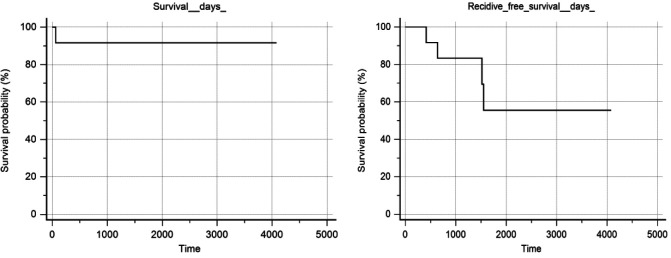
Kaplan-Meier analysis of the overall survival and progression-free survival of liver transplanted patients with neuroendocrine tumors in Merkur University Hospital.

None of the 12 patients received peritransplant treatment. Kaplan-Meier analysis was performed to estimate the overall survival (OS) and progression-free survival (PFS). In the follow-up period of 60-4079 days, 11 of 12 patients were alive (OS 91.67%). At 638-4079 days, eight 8 of 12 patients were alive without signs of recurrence (PFS 66.67%) (Figure 1). One patient died after LT due to hepatic artery thrombosis. In the post-transplant period (17-48 months after LT, median 19 months), disease recurrence was observed in four patients (36.36%).

## Discussion

During the past few decades, LT has evolved from a high-risk treatment to a routine operation with a good survival outcome of >80% at 1 year and >70% at 5 years (21-23). In addition, LT for NET liver metastasis showed a high survival and disease-free rate (40%) (6,24), although a relatively low number of LTs was performed in these patients, and the tumors showed a highly variable behavior.

NET heterogeneity with inconsistent patient selection criteria lead to a wide range of five-year survival (33%-97%) and disease-free survival (DFS) rates (32%-87%) (3,6,8,12,13,17,20,25-32), considerably varying according to tumor grade, stage, primary site, age, and period from diagnosis, with reported improvement of median overall survival (9.3 years) (32)

There is no literature consensus regarding the difference in LT prognosis between gastrointestinal NETs (traditionally referred to as carcinoid) and pancreatic islet cell tumors. LT is usually indicated in patients with tumors whose primary location is drained mainly by the portal system and that more often produce only hepatic metastases (6,14). The research group that first implemented this prerequisite offered transplantation only to the patients with primary tumor originating in the pancreas and from the distal stomach to sigmoid colon, achieving respectable results (6). Patients with carcinoid tumors were suggested to have better LT results due to a slower growth rate, better response to chemoembolization, and more frequent localization in the midgut, with lower surgical risk (3,12,13,33). A recent retrospective, population-based study revealed increased OS in distant stage gastrointestinal and pancreatic NET due to the use of novel agents and universal tumor staging and grading (12,32,34-38). An analysis of the UNOS database by Gedaly et al (28) revealed no significant survival difference between carcinoid and non-carcinoid group.

Only a few cases of LT in patients with primary NET of the liver have so far been reported in the literature, preventing any conclusions on that tumor localization (39-42). These tumors are suggested to have a better prognosis than secondary hepatic NETs, with long disease-free periods and survival in liver recipients. Nevertheless, when indicating LT in these patients a detailed diagnostic work-up is required to exclude an extrahepatic primary tumor, especially with the advent of novel, sensitive diagnostic tools.

Apart from the sole presence of liver metastases, Frilling et al emphasized the significance of their localization pattern (43). They described three types of metastatic spread in the liver, proposing a connection between localization type and tumor biological behavior. A single metastasis of any size (type I) was noted in 19.3% of patients; isolated metastatic bulk accompanied with multiple deposits, with both lobes involved (type II), was noted in 15.1% of patients; and disseminated metastatic spread with both lobes involved, single lesion of varying size, and no normal parenchyma (type III) was noted in the majority of patients (65.5%). Only type-I patients were candidates for surgical resection, while the patients with type II-III were LT candidates.

Although the usual prerequisite for LT is ≤50% liver hepatic involvement, Olausson et al performed LT in patients with large tumor burden, achieving respectable five-year survival of 90% (6,44,45). According to the authors, these remarkable results may be attributed to the strict follow-up protocol and active recurrence treatment.

Being the basis of the grading scheme adopted by the latest WHO classification, the Ki-67 proliferation index was widely evaluated in respect to LT outcome (26,29,46-48). An analysis of Ki-67 and E-cadherin, as an indicator for metastatic potential, revealed their significance in survival prediction (26). Intra-tumoral heterogeneity, causing discrepant proliferation rates, was reported in nearly 50% of cases (49,50).

Ever since LT was first performed in patients with NETs, attempts have been made to achieve the results similar to those in patients with benign liver disease. Due to donor organ scarcity and persistent ethical issues, there was an effort to create standardized selection criteria and justify LT for this group of patients with malignant disease (1,6,17,18).

The deleterious effect of disease recurrence was already noted by Le Treut et al, who reported a poor five-year survival of 36% and a DFS of 17% (12). Consequently, a prerequisite of R0 primary tumor resection and meticulous exclusion of extrahepatic disease were implemented as the criteria for LT candidacy (6,9,17,18).

With the advent of somatostatin receptor scintigraphy (SRS) and especially novel, more sensitive positron emission tomography in combination with computed tomography (PET/CT), the exclusion of extrahepatic disease has significantly contributed to the overall LT success (1,30,51). The use of more sensitive and highly specific somatostatin analogues (DOTANOC, DOTATOC, DOTATATE) in PET/CT has improved imaging up to 30% compared with standard modalities (1). Mojtahedi et al (51) showed that ^68^Ga-DOTATATE PET/CT compared with SRS changed the clinical management of 70.6%-81% patients with NETs.

Along with the presence of unresectable hepatic metastases with well-differentiated primary tumor and no extrahepatic disease as universally accepted conditions for LT, many other factors were listed in the constantly evolving selection criteria. In 2016, Mazzaferro et al presented the Milan criteria (6). These strict and very selective criteria attributed to the excellent five-year survival of 97% and DFS of 87% observed in their study. They included patients <60 years of age with histologically confirmed low grade G1-G2 NET, tumor drained primarily by the portal system, a good response and disease stability of at least six months during the pre-transplantation period, with all extrahepatic sites with ≤50% liver involvement completely removed.

Following a scientific consensus, several recommendations for patient selection for LT were presented (Table 2). Transplantation was advised in operable patients with unresectable symptomatic or asymptomatic disease, the disease confined to the liver, a well-differentiated primary tumor, and without associating major extrahepatic resections to LT.

**Table 2:** Selection criteria on liver transplantation (LT) for pancreatic neuroendocrine tumor liver metastases. Adapted from (20)

	Milan criteria 2016 (6)	UNOS guidelines 2017 (38)	ENETS guidelines 2016 (37)
Histology grade†	G1–G2	G1–G2	G1–G2
Primary tumor site	Portal system drainage	Portal system drainage	NA
Tumor involvement	<50% of the liver volume	<50% of the liver volume	NA
Time interval of stable disease between primary tumor resection to LT	Resection of primary tumor and all extra-hepatic tumor deposits and stable disease/good response to therapies for at least 6 months	Resection of primary malignancy and extra-hepatic disease without any evidence of recurrence at least 6 months	NA
Recipient age	<60 years	<60 years	NA
Other	Extended Milan criteria <70 years	GEP origin Neuroendocrine liver metastasis restricted to the liver, bi-lobar, not amenable to resection Negative meta workup	Functional NETs and diffuse liver disease, early refractory to multiple systemic treatment; exclusion of extrahepatic disease; low bilirubin; carcinoid syndrome or functional NETs

*Abbreviations: UNOS – United Network for Organ Sharing; ENETS – European Neuroendocrine Tumor Society; NA – not applicable; GEP – gastro-entero-pancreatic

†World Health Organization Classification of Neuroendocrine Tumors 2010.

In some studies a special attention was given to diagnosis-to-LT time interval (3,6,18,25,29,30,32,52), although it has not been proven as a significant prognostic factor in univariate analysis. Interestingly, doubling of its median value was accompanied by an improvement in the OS, indicating that LT should be offered to asymptomatic patients only after the disease became refractory to other therapeutical modalities.

The optimal timing of LT has been critically evaluated in several studies (3,6,18,25,29,30,32,52). While some authors proposed LT in patients with a stable disease and good response to therapy, others advocated it only in symptomatic patients refractory to other types of treatment. The conclusion was that the existing data are insufficient for final recommendations, warranting prospective analyses, including those assessing disease stability and treatment response (17).

Recently, Norlén et al (53) have published an interesting study on 78 patients with NET treated with therapeutic modalities other than LT. The study enrolled patients <65 years of age, with successful primary tumor removal and liver metastases, but without extrahepatic disease. They were treated with liver resection, ablation, and hepatic artery embolization, as well as with somatostatin analogues and interferon alpha. The research obtained an excellent 84 ± 8% and 92 ± 9% five-year survival for patients <65 and <55 years of age, respectively. The group (n = 33) fulfilling the Milan criteria had 97 ± 6% five-year survival, which supports the thesis that the excellent result from the Milan series may be attributed to selection bias rather than to treatment effect. Based on these favorable survival results, the authors emphasized the need for randomized controlled trials and further analysis of survival after LT without the use of narrow selection criteria. When properly indicated, LT could be a potentially curative option, sparing some patients from unnecessary operation risk and life-long immunosuppression burden.

This study found high-grade NET (especially neuroendocrine cancer) and primary tumor in the pancreas to be the main poor prognostic factors, with a mortality rate 8.3% due to surgical complications. Age, sex, tumor functional status, and Ki-67 did not significantly affect the disease recurrence. We cannot confirm the prognostic significance of aggressive metastatic NET and the time interval between primary tumor resection and LT.

Being one of only a few metastatic disease indications for LT, NETs were widely evaluated in the literature. Despite the recognition of several prognostic factors and selection protocols, clear recommendations for routine clinical practice have not yet been fully developed due to disease rarity and variable biologic behavior. With the advent of new and sophisticated diagnostic modalities, prospectively evaluated selection criteria, and the evaluation of multimodal therapeutic options within multidisciplinary teams, LT should be increasingly referred to as a long-term cure for this group of patients.
